# Comprehensive circRNA Analyses in Human Vertebrae of GIOP and Its Molecular Mechanism

**DOI:** 10.1155/2022/4203161

**Published:** 2022-02-08

**Authors:** Linfeng Wang, Hong Ye, Douquan Huang, Chengwu Lu, Weiming Lin, Xiaojie Chen

**Affiliations:** ^1^Department of Orthopedics, the Affiliated Nanping First Hospital of Fujian Medical University, Nanping 353000, Fujian, China; ^2^Department of Orthopedics, the People's Hospital of Nanping, Nanping, Fujian, China

## Abstract

Circular RNAs (circRNAs) are a novel class of noncoding RNAs that play important roles in human diseases. However, the regulation of circRNAs in glucocorticoid-induced osteoporosis (GIOP) has not been reported. In this study, we performed high-throughput sequencing to identify altered circRNAs in the vertebrae from GIOP patients. A total of 65 clinical samples were collected in this study. Bioinformatics algorithms were employed to predict the target relationship between circRNAs and miRNAs and the circRNAs-miRNAs regulatory network. We focused on the top 10 significantly up-/downregulated circRNAs (hsa_circ_0004906, hsa_circ_0001172, hsa_circ_0005778, hsa_circ_0004276, hsa_circ_0005729, hsa_circ_0006173, hsa_circ_0007662, hsa_circ_0001451, hsa_circ_0001564, and hsa_circ_0108735) and measured their expression by qRT-PCR in clinical samples. Bioinformatics analyses demonstrated that 87 miRNAs were predicted in upregulated circRNAs and 104 miRNAs were predicted in downregulated circRNAs. The functional enrichment analysis showed these targeted miRNAs were significantly enriched in bone metabolism-related biological processes and pathways, including the MAPK signaling pathway, positive regulation of the metabolic process and metabolic pathways, etc. Collectively, our study revealed circRNA regulation and circRNAs-miRNAs regulatory network in GIOP for the first time, which provides a new perspective on the molecular mechanism of GIOP and lays a foundation for GIOP treatment.

## 1. Introduction

Glucocorticoid-induced osteoporosis (GIOP) is the most common form of secondary osteoporosis induced by the long-term use of glucocorticoids (GCs) [1–3]. Emerging evidence has shown that the disability and mortality rates of GIOP are much higher than those of common osteoporosis [3]. Pathophysiologic mechanisms leading to GIOP work in concert to increase bone fragility through reducing bone formation and increasing bone resorption [4, 5]. However, the underlying mechanism of GIOP has not been fully elucidated, and effective treatment strategies are urgently needed.

Circular RNAs (circRNAs) represent a novel type of noncoding RNAs that are formed by the circularization of back-splicing events [6, 7]. circRNAs are widely expressed in humans in a tissue- or cell-type-specific manner. Many studies have found that circRNAs could act as sponges of microRNAs (miRNAs) and competitively suppress miRNA activity to regulate gene expression [8, 9]. The association between human diseases and altered circRNA levels has also been investigated. Accumulating evidence showed that circRNAs are potential diagnostic biomarkers for human diseases [10]. However, the global expression and potential mechanism of circRNAs in GIOP remain largely unknown.

To better understand the molecular mechanisms of GIOP, we performed high-throughput sequencing and qRT-PCR in GIOP vertebrae to identify circRNA expression profiles for the first time. Moreover, we generated the target miRNAs based on bioinformatics algorithms and built a circRNAs-miRNAs regulatory network. Furthermore, Gene Ontology (GO) and Kyoto Encyclopedia of Genes and Genomes (KEGG) analyses were performed to elucidate the biological significance of notably altered circRNAs, which provide a new perspective on the molecular mechanism of GIOP and lay a foundation for GIOP treatment.

## 2. Materials and Methods

### 2.1. Clinical Samples

A total of 65 clinical samples were collected from thirty-seven GIOP patients and twenty-eight healthy volunteers between October 2015 and January 2019 at the Orthopedics Department of The Affiliated Nanping First Hospital of Fujian Medical University. Six pairs of GIOP samples and control samples were used for high-throughput sequencing, while another thirty-one GIOP samples and twenty-two control samples were used for experimental validation. The diagnostic criteria for GIOP patients have characterized the long-term GC use (more than 6 months) and osteoporosis/osteopenia (T-score ≤ −2.5/T-score > −2.5 and <−1.0). Postmenopausal women, patients suffering from other diseases that may cause osteoporosis (tumor, hyperparathyroidism, diabetes, etc.), and patients receiving any antiosteoporosis treatment were not included in this study. The inclusion criteria for the control group were as follows: (a) No metabolism diseases that might affect bone metabolism. (b) No osteoporosis and history of GC use. (c) Non-postmenopausal women. The clinical information (age, gender, body mass index, T-score, etc.) was collected from all participants. The vertebral bone samples were collected by bone biopsy from two groups.

### 2.2. Institutional Ethics Statement

This study was approved by the Ethics Committee of The Affiliated Nanping First Hospital of Fujian Medical University and was in accordance with the Helsinki Declaration. All participants were well informed of the study protocol, and written informed consent was signed by all participants.

### 2.3. Library Construction and High-Throughput Sequencing

After grinding the vertebral bone tissue in liquid nitrogen condition, the bone powder was collected for RNA isolation. Total RNA was extracted by TRIzol (Invitrogen, CA, USA). RNA concentration and integrity were evaluated by using the Qubit RNA Assay Kit (Invitrogen, CA, USA) and Agilent 2100 Bioanalyzer (Agilent Technologies, CA, USA), respectively, with acceptance criteria of 28S : 18S ratio ≥ 1.5 and RNA integrity number (RIN) ≥  7.0.

Subsequently, total RNA was digested with RNaseR to remove linear RNAs. The cDNA library was constructed according to the manufacturer's guidance, and then, the library was subjected to paired-end Illumina sequencing (Illumina, CA, USA).

### 2.4. Expression Analysis of circRNA-SeqData

The raw sequencing data were filtered by Fast QC and NGSQC software to obtain high-quality clean reads [11]. Subsequently, the clean reads were mapped to human reference genome Hg38 by the Top Hat tool with default parameters. Next, the unmapped reads were subjected to CIRI, CIRC explorer, and find_circ software to identify circRNAs [12]. The circRNA annotation was based on the circbase database [13]. Differentially expressed circRNAs were analyzed by the RLimma package with threshold of |fold change| >2 and *P* value < 0.05.

### 2.5. circRNAs-miRNAs Interaction Analysis

The potential target miRNAs for circRNAs were predicted by RNAhybrid, miRanda, and TargetScan software with default parameters [14]. The Venn diagram shows the number of target miRNAs predicted by the three software. The circRNAs-miRNAs interaction network was built by Cytoscape software.

### 2.6. GO and KEGG Analyses

Gene Ontology and KEGG pathway analyses enable us to describe gene attributes and regulatory mechanisms in humans. The function of miRNAs targeted by circRNAs was investigated by the miRPathv.3.0 tool [15]. The *P* value represents the enrichment significance of GO terms and pathways, and *P* value < 0.05 was considered as statistically significant.

### 2.7. Quantitative Real-Time PCR (qRT-PCR) Analysis

QRT-PCR experiments were performed to verify the expression of circRNAs in GIOP samples and control samples. Specific primers were designed by Sangon Biotech (Shanghai, China), which span the back-splice junction region of circRNAs. Total RNAs were transcribed to cDNAs using the PrimeScripRT reagent Kit (TaKaRa, China) following the instruction. PCR analyses were performed using a real-time PCR system using the SYBR Green PCR kit (Takara, Japan). GAPDH was used as internal control, and the relative expression level was calculated by the 2^−ΔΔCt^ method.

The sequences of primers were as follows:

- hsa_circ_0001451: sense, 5′-CAACAAAAGAUUACUUCCUTT-3′, antisense, 5′- AGGAAGUAAUCUUUUGUUGTT-3′
- hsa_circ_0007662: sense, 5′-TGTGGGGGAAAAACAGGGTT-3′, antisense, 5′- ACGAGAAATGACAAGAGTAGCTGA-3′
- hsa_circ_0006173: sense, 5′-CCAGACAGGACTTTCTTCTGCT-3′, antisense, 5′-TGTGAGATCTCCATGGGCTGA-3′
- hsa_circ_0001564: sense, 5′- CATCCTTTGCGCTCAGAGGA-3′, antisense, 5′- GATTGGCCTGACCACAGTCTA-3′
- hsa_circ_0108735: sense, 5′- GCTTCTCCAGGCCAGACATT-3′, antisense, 5′- GCTGCTGTGGTTGTTTCTGG3′
- hsa_circ_0004276: sense, 5′-GCTCACAGCTGATCCTAAGGT-3′, antisense, 5′-GACGTTGGTTCCTTCAAGCC-3′
- hsa_circ_0001172: sense, 5′- ACAAAGCCCAGATCCAGGTG-3′, antisense, 5′- GTATCGACAGTCTGGGCTCG-3′
- hsa_circ_0005729: sense, 5′- CAATGCCAAGACAGAGCTGC-3′, antisense, 5′- GCTTTCCTCGAGCTTCCTGT3′
- hsa_circ_0005778: sense, 5′- CATCCTTTGCGCTCAGAGGA-3′, antisense, 5′- GATTGGCCTGACCACAGTCTA-3′
- hsa_circ_0004906: sense, 5′- AGTTGCGCTCCCAATCTCTC-3′, antisense, 5′- GTCTCGGTCCGTTACACCAG-3′
- GAPDH: sense, 5′-CATGGGTGTGAACCATGAGA-3′, antisense, 5′-CAGTGATGGCATGGACTG-TG-3′

## 3. Results

### 3.1. General Information of Study Subjects

Thirty-seven clinically stable patients with GIOP (21 males and 16 females) and twenty-eight healthy control subjects (16 males and 12 females) were enrolled in this study. As shown in Table 1, there were no significant differences in age, Body Mass Index (BMI), and serum Mg/P/ALP levels between the patient group and control group, while BMD, T-score, and serum Ca level were significantly lower in the patient group than in the control group (*P* value < 0.05).

### 3.2. Expression Profiles of circRNAs in GIOP

**Figure 1:**
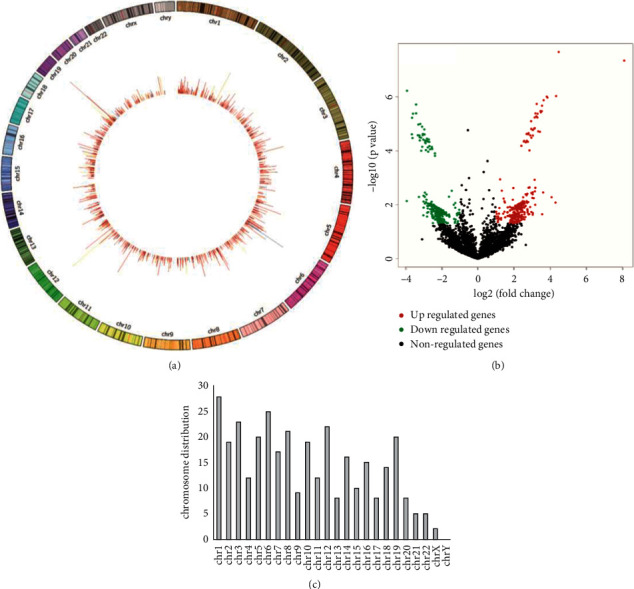
circRNA expression in GIOP.(a) Chromosome distribution of detected circRNAs. (b) Differentially expressed circRNAs in GIOP. Red: upregulated. Green: downregulated. (c) Chromosome distribution of differentially expressed circRNAs.

The high-quality clean reads were obtained from six pairs of GIOP samples and control samples by using the Illumina Hiseq sequencer. A total of 17,348 circRNAs were detected by sequence alignment in the Hg38 genome and circbase database. These circRNAs were unevenly distributed in human chromosomes except for the sex chromosome Y (Figure 1(a)). By filtering the fold change and *P* value (|fold change| >2 and *P* value < 0.05), we identified 338 circRNAs that are differentially expressed between two groups. A volcano plot of the differentially expressed circRNAs is shown in Figure 1(b). The chromosome distribution of differentially expressed circRNAs was also analyzed, and the results showed that most of the circRNAs were distributed on chr1, chr6, and chr12 (Figure1(c)). The top 10 dysregulated circRNAs are listed in Table 2, including hsa_circ_0004906, hsa_circ_0001172, hsa_circ_0005778, hsa_circ_0004276, hsa_circ_0005729, hsa_circ_0006173, hsa_circ_0007662, hsa_circ_0001451, hsa_circ_0001564, and hsa_circ_0108735.

### 3.3. Target miRNA Prediction

**Figure 2:**
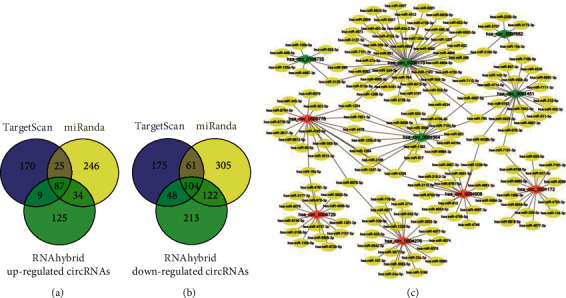
circRNAs-miRNAs regulatory network.(a), (b) Predicted miRNAs in up- and downregulated circRNAs by three algorithms. (c) circRNAs-miRNAs regulatory network was built by Cytoscape.

Studies have shown that circRNAs may act as sponges of miRNAs to regulate gene expression. Therefore, we predicted the target miRNAs of the top 10 dysregulated circRNAs by bioinformatics tools. There were 679, 984, and 742 miRNAs predicted by TargetScan, miRanda, and RNAhybrid algorithms, respectively. The intersection results of the three bioinformatics algorithms demonstrated that 87 miRNAs were predicted in upregulated circRNAs and 104 miRNAs were predicted in downregulated circRNAs (Figures 2(a) and 2(b)).

### 3.4. circRNAs-miRNAs Network Analysis

As shown in Figure 2(c), a circRNAs-miRNAs interaction network was built for the top 10 dysregulated circRNAs to investigate the interaction between circRNAs and target miRNAs in GIOP. For the upregulated circRNAs, hsa_circ_0004276 and hsa_circ_0005778 had more cross links with target miRNAs than other circRNAs. In the downregulated circRNAs, the most cross-linked interactions with target miRNAs were hsa_circ_0006173 and hsa_circ_0001451.

### 3.5. Prediction of the Characteristics of circRNAs Related to GIOP Using GO and KEGG Analyses

**Figure 3:**
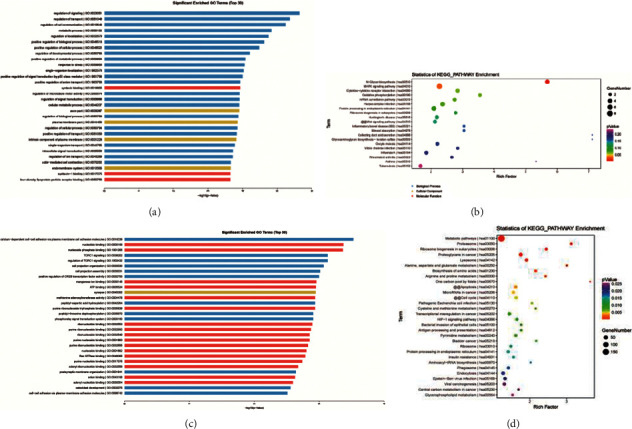
GO and KEGG analyses for targeted miRNAs. (a), (b) MiRNAs targeted by upregulated circRNAs. (c), (d) MiRNAs targeted by downregulated circRNAs.

To further investigate the biological roles of circRNAs-miRNAs interaction in GIOP, we performed GO and KEGG analyses of target miRNAs using DIANA-miRPathv.3.0 software, which systematically collects the experimentally validated miRNA target genes and their functions. The results showed miRNAs targeted by upregulated circRNAs were mainly enriched in the regulation of signaling/transport, metabolic process, N-glycan biosynthesis, MAPK signaling pathway, and cytokine-cytokine receptor interaction (Figures 3(a) and 3(b)). The miRNAs targeted by downregulated circRNAs were mainly enriched in calcium-dependent cell-cell adhesion via plasma membrane cell adhesion molecules, nucleotide binding, TORC1 signaling, metabolic pathways, and alanine metabolism (Figures 3(c) and 3(d)). The abovementioned findings indicated that the top 10 dysregulated circRNAs may play key roles in GIOP by interacting with specific miRNAs.

### 3.6. Validation of circRNAs by qRT-PCR

**Figure 4:**
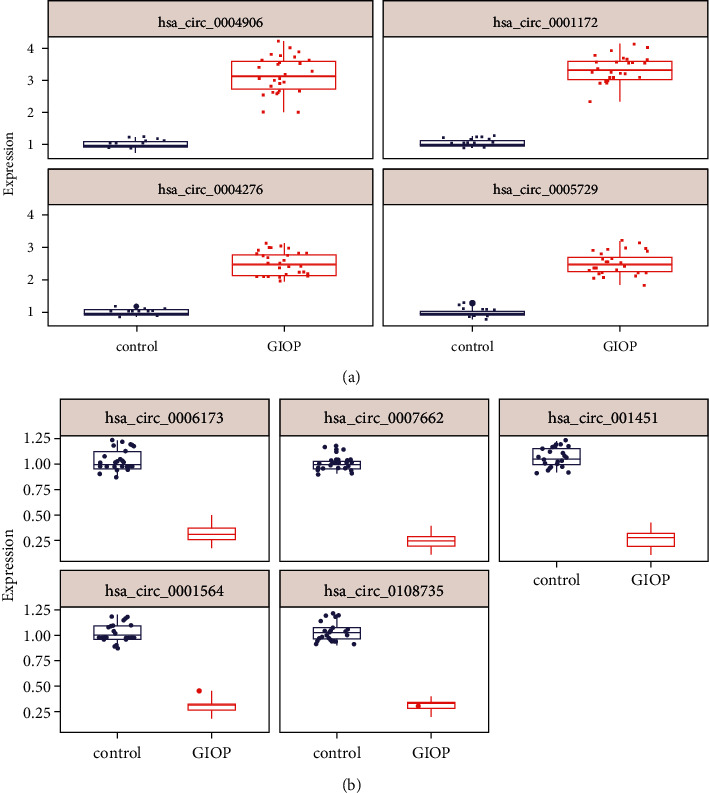
Validation of circRNAs by qRT-PCR. (a) Four upregulated circRNAs. (b) Five downregulated circRNAs. *P* value < 0.05.

To verify the expression of the top 10 dysregulated circRNAs, we performed qRT-PCR experiments in another thirty-one GIOP samples and twenty-two healthy control samples. The results showed that hsa_circ_0004906, hsa_circ_0001172, hsa_circ_0004276, and hsa_circ_0005729 were significantly upregulated in GIOP samples while hsa_circ_0006173, hsa_circ_0007662, hsa_circ_0001451, hsa_circ_0001564, and hsa_circ_0108735 were significantly downregulated (Figures 4(a) and 4(b), *P* value < 0.05), consistent with high-throughput sequencing data. However, the expression of hsa_circ_0005778 did not show an obvious decrease in GIOP samples, which is different from sequencing data (fold change: 1.35; *P* value = 0.19; data not shown).

## 4. Discussion

In recent years, circRNAs have emerged as a novel class of endogenous RNAs dysregulated in human tissues [9]. circRNAs are potential ideal diagnostics biomarkers for diseases because of their special loop structures and characteristics [6, 10]. Several studies have shown that dysregulated circRNAs are associated with bone-related diseases such as osteoporosis [16–18]. However, little is known about the global expression and potential role of circRNAs in GIOP.

In the present study, we first analyzed the circRNA expression profiles in GIOP patients by high-throughput sequencing and bioinformatics analyses. Our results showed that 338 circRNAs were significantly differentially expressed between the GIOP group and control group, suggesting their important roles in GIOP pathophysiology. We also found that these differentially expressed circRNAs were widely distributed on each chromosome except for the Y chromosome.

The top 10 significantly up-/downregulated circRNAs in GIOP were further investigated, including hsa_circ_0004906, hsa_circ_0001172, hsa_circ_0005778, hsa_circ_0004276, hsa_circ_0005729, hsa_circ_0006173, hsa_circ_0007662, hsa_circ_0001451, hsa_circ_0001564, and hsa_circ_0108735. By means of bioinformatics tools, we identified several miRNAs that have binding sites with circRNAs in their sequences. It is worth noting that many of the targeted miRNAs were revealed to participate in bone-related diseases, including hsa-miR-125b-2-3p, which is a key regulator in mediating chemotaxis and survival of bone marrow-derived granulocytes [19]. hsa-miR-450b could promote osteogenic differentiation and bone formation [20]. miRNAmir-503-5p was found to regulate chondrocyte proliferation and hypertrophic differentiation in rats [21]. Regulation of miR-96-5p may affect cell proliferation during craniofacial and dental development [22]. The circRNAs-miRNAs interaction network demonstrated that the top 10 significantly up-/downregulated circRNAs may act as sponges of the abovementioned bone-relevant miRNAs, indicating their potential roles in bone development.

We next performed GO and KEGG analyses for the miRNAs in the network and found that the enriched functional terms are related to GIOP, for example, metabolic pathways and N-glycan biosynthesis, which are important pathways in bone metabolism [23]. Other important biological processes including calcium-dependent cell-cell adhesion via plasma membrane cell adhesion molecules and positive regulation of metabolic process were also involved [23, 24]. Finally, the expression of the top 10 significantly up-/downregulated circRNAs was verified by qRT-PCR experiments in thirty-one GIOP samples and twenty-two healthy controls. We noticed that the expression of hsa_circ_0005778 was inconsistent with sequencing data; this may due to the limitation of sample size [25]. It has been reported that hsa_circ_0002060 depletion attenuates osteoporosis by regulating miR-198-5p[26]. circRNA_0006393 contributes to osteogenesis by targeting miR-145-5p/FOXO1 in glucocorticoid-induced osteoporosis [27]. There were still some limitations in the current study. For example, the specific function of these circular RNAs in GIOP development should be explored in future investigations. Whether the differential circRNAs can form a loop should be verified in the future. The stability of circRNAs needs to be validated by more experiments.

In summary, our study revealed the expression profiles of circRNAs in GIOP by high-throughput sequencing and qRT-PCR validation for the first time and identified several significantly up-/downregulated circRNAs that may act as candidate regulatory molecules for GIOP development. Moreover, the circRNAs-miRNAs regulatory network and related functional enrichment were systematically investigated, new perspectives on the molecular mechanism of GIOP were provided, and the base for GIOP treatment was established.

## Figures and Tables

**Table 1:** Clinical information of GIOP patients and healthy controls.

Categories (mean ± SD)	GIOP	Control	*P* value
Age	57.20 ± 6.01	56.90 ± 6.23	-
BMI (kg/m^2^)	20.31 ± 1.82	21.02 ± 1.75	-
BMD (g/cm2)	0.54 ± 0.06	1.03 ± 0.10	0.001
T-score	−3.9 ± 0.20	−0.3 ± 0.95	<0.001
Serum Ca (mmol/L)	1.89 ± 0.07	2.23 ± 0.09	0.009
Serum P (mmol/L)	1.05 ± 0.05	1.18 ± 0.16	-
Serum ALP (U/L)	69.8 ± 16.88	65.97 ± 19.38	-
Serum Mg (mmol/L)	0.77 ± 0.09	0.80 ± 0.10	-

**Table 2:** Top 10 dysregulated circRNAs in GIOP samples compared to control.

circRNA	Log_2_FC	*P* value	Regulation
hsa_circ_0001451	−3.92403434923793	0.00725696428732379	Down
hsa_circ_0007662	−3.90949282452378	5.83222151202752E-07	Down
hsa_circ_0006173	−3.64206081838854	0.0000240629909097923	Down
hsa_circ_0001564	−3.62806417075557	5.90118623461766E-06	Down
hsa_circ_0108735	−3.60470408758295	0.0000329665851665041	Down
hsa_circ_0004276	7.87499859877847	2.14799948992582E-08	Up
hsa_circ_0001172	4.92789131907627	9.21641505754447E-07	Up
hsa_circ_0005729	4.69595826779837	0.00830055155874856	Up
hsa_circ_0005778	4.34828670752697	1.04632586419008E-06	Up
hsa_circ_0004906	4.10986276586592	9.65227226638277E-07	Up

## Data Availability

The datasets used and/or analyzed during the current study are available from the corresponding author on reasonable request.
